# Successful Closure of Multiple Post-bariatric Surgery Fistulas Using Modified Internal and External Endoscopic Vacuum Therapy: A Case Report

**DOI:** 10.7759/cureus.105754

**Published:** 2026-03-24

**Authors:** John A Lata Guacho, Marcelo Simas de Lima, José A Sánchez León, Carlos A Ruiz Sosa, Janneth A Valdivieso Maggi, Mauricio N Suárez Caicedo

**Affiliations:** 1 Gastroenterology, Eugenio Espejo Specialty Hospital, Quito, ECU; 2 Gastroenterology, University of São Paulo, São Paulo, BRA; 3 Digestive Endoscopy, University of São Paulo, São Paulo, BRA; 4 Oncological Endoscopy, Institute of Cancer of São Paulo, São Paulo, BRA; 5 Gastroenterology, Pontifical Catholic University of Ecuador, Quito, ECU; 6 Gastroenterology, Institute of Cancer of São Paulo, São Paulo, BRA; 7 Bariatric and Metabolic Surgery, Central University of Ecuador, Quito, ECU

**Keywords:** bariatric surgery, case reports, digestive system fistula, endoscopy, negative-pressure wound therapy

## Abstract

Leaks and fistulas following bariatric surgery are serious complications associated with sepsis, malnutrition, and high morbidity. Endoscopic vacuum therapy (EVT) has emerged as an effective minimally invasive alternative for the management of transmural defects of the upper gastrointestinal tract. Recently, low-cost modifications using conventional hospital supplies have been developed to expand their applicability in resource-limited settings. We present the case of a 46-year-old female patient with a history of multiple bariatric surgeries: sleeve gastrectomy (2017), Roux-en-Y gastric bypass (2019), and revisional surgery in 2024 for candy cane syndrome. In the immediate postoperative period, she developed necrotizing acute pancreatitis and septic shock secondary to gastric pouch leakage, requiring multiple laparotomies. She evolved with three fistulous tracts: one gastrocutaneous and two enterocutaneous, confirmed by gastrointestinal transit, contrast-enhanced tomography, and upper gastrointestinal endoscopy. Endoscopic management was performed using modified internal and external EVT constructed with a nasogastric tube, gauze, and antimicrobial dressing. Initially, an external system was placed under a hydrophilic guide through the fistulous tract. Subsequently, after overcoming jejunal stenosis with an ultrathin endoscope, an internal modified system was placed. Both systems were connected to continuous suction at 125 mmHg. After 29 days of treatment, clinical, endoscopic, and radiologic closure of the fistulous tracts was achieved. The patient showed favorable evolution, recovery of oral tolerance, and was discharged in good general condition. In conclusion, modified EVT, both internal and external, represents an effective and reproducible alternative for managing complex post-bariatric surgery fistulas. Its adaptation using low-cost hospital supplies may expand access to this technique in resource-limited healthcare systems, offering favorable outcomes in high-complexity scenarios.

## Introduction

Bariatric surgery comprises a group of procedures that induce weight loss through restrictive and metabolic mechanisms and represents the most effective treatment for obesity and its associated complications [1,2]. In 2022, extensive evidence on its safety and long-term positive effects led the International Federation for the Surgery of Obesity and Metabolic Disorders (IFSO) and the American Society for Metabolic and Bariatric Surgery (ASMBS) to update the indications for these procedures, originally published in 1991 by the United States National Institutes of Health. The most common bariatric procedures are sleeve gastrectomy (SG) and Roux-en-Y gastric bypass (RYGB) [2,3].

Among complications following bariatric surgery, gastrointestinal transmural defects stand out, defined as full-thickness rupture of the gastrointestinal wall, classified into leaks, perforations, and fistulas; leaks and fistulas significantly increase morbidity and mortality, especially in the acute stage [4,5].

Leaks after SG may lead to fistula formation, with an incidence ranging from 0.2% to 2.5%, most frequently located in the upper third of the gastroplasty [6,7]. Leaks are also major complications after RYGB, occurring in 0.7% to 5% of patients, commonly located at the gastrojejunal anastomosis, but also described in the distal esophagus, gastric pouch, blind jejunal limb, and jejunojejunal anastomosis [8,9].

Among available therapeutic options for leaks and fistulas, the endoscopic approach has proven effective in appropriately selected patients [10]. One of the therapies with the highest closure rates and lowest complication and mortality rates is the endoscopic vacuum therapy (EVT), which is a minimally invasive endoscopic technique designed for the treatment of gastrointestinal transmural defects such as leaks, perforations, and fistulas. The technique involves the endoscopic placement of a drainage device connected to a suction system directly at the site of the defect. Typically, a porous material such as a polyurethane sponge or gauze is attached to a nasogastric or drainage tube and positioned either within the gastrointestinal lumen or inside the associated extraluminal cavity under endoscopic guidance. Continuous negative pressure is then applied through the catheter, usually between 75 and 125 mmHg, allowing active drainage of infected secretions, collapse of the cavity, reduction of bacterial contamination, improvement of tissue perfusion, and stimulation of granulation tissue formation. Treatment duration varies according to the size of the defect, the presence of infection, and the patient’s clinical status, but closure is often achieved within several weeks of therapy [11-13]. Studies have shown a 21% higher closure rate compared with self-expanding metal stents (SEMS), along with a 12% reduction in mortality and a mean reduction of 14.22 days in treatment duration, as well as a 24% decrease in adverse events [14].

Although polyurethane sponge is typically used in EVT, the endoscopic approach presents challenges such as placement difficulty and the need for multiple exchanges, increasing costs. Therefore, modified EVT techniques have been developed using commonly available hospital materials such as nasogastric tubes, gauze, and antimicrobial surgical drapes to create a cost-effective and accessible system that can be implemented in resource-limited settings [15].

Based on EVT developed by Brazilian researchers, to the best of our knowledge, we present the first reported case in Ecuador of successful fistula closure using modified internal and external EVT.

## Case presentation

A 46-year-old female patient with a history of SG performed in 2017 developed de novo gastroesophageal reflux disease, attributed to an undiagnosed hiatal hernia with severe symptoms. In 2019, conversion to RYGB was performed via an open approach due to extensive intra-abdominal adhesions that prevented laparoscopic treatment.

Due to the persistence of symptoms characterized by abdominal pain, nausea, and late vomiting, in 2024, she was diagnosed with “candy cane” syndrome, a postoperative complication of RYGB characterized by the presence of an excessively long blind afferent limb proximal to the gastrojejunal anastomosis. This redundant intestinal segment can cause food stasis and progressive dilation of the blind limb, leading to symptoms such as abdominal pain, nausea, vomiting, reflux, and food intolerance [16]. The diagnosis was established by simple and contrast-enhanced thoracoabdominal tomography, after which revisional bypass surgery, hiatoplasty, and concomitant cholecystectomy were performed, reducing the gastric pouch from 80 cc to 30 cc and resection of the redundant limb.

On the first postoperative day, the patient presented with tachycardia and severe abdominal pain, leading to suspicion of an acute abdomen secondary to possible anastomotic leakage. Due to clinical suspicion, another exploratory laparotomy was performed, revealing saponification foci in the omentum and peritoneum consistent with necrotizing acute pancreatitis, for which abdominal lavage and drain placement were performed.

Subsequently, the clinical course remained unfavorable, developing an intra-abdominal collection secondary to gastric pouch leakage and progression to septic shock. Another exploratory laparotomy was performed with drainage of the collection and culture sampling, which was positive for *Klebsiella pneumoniae* producing extended-spectrum beta-lactamases (ESBL). The leakage site was treated with the placement of a Kehr T-tube. This clinical course highlights several warning signs that may suggest postoperative complications such as leaks or fistulas following bariatric surgery. Early postoperative tachycardia, severe abdominal pain, persistent sepsis, and abnormal drainage from surgical wounds are recognized clinical red flags that should prompt early diagnostic evaluation in patients with recent bariatric procedures. In this case, the persistence and progression of these findings raised suspicion of an underlying gastrointestinal defect, which prompted further diagnostic evaluation and multidisciplinary management [17,18].

**Figure 1:**
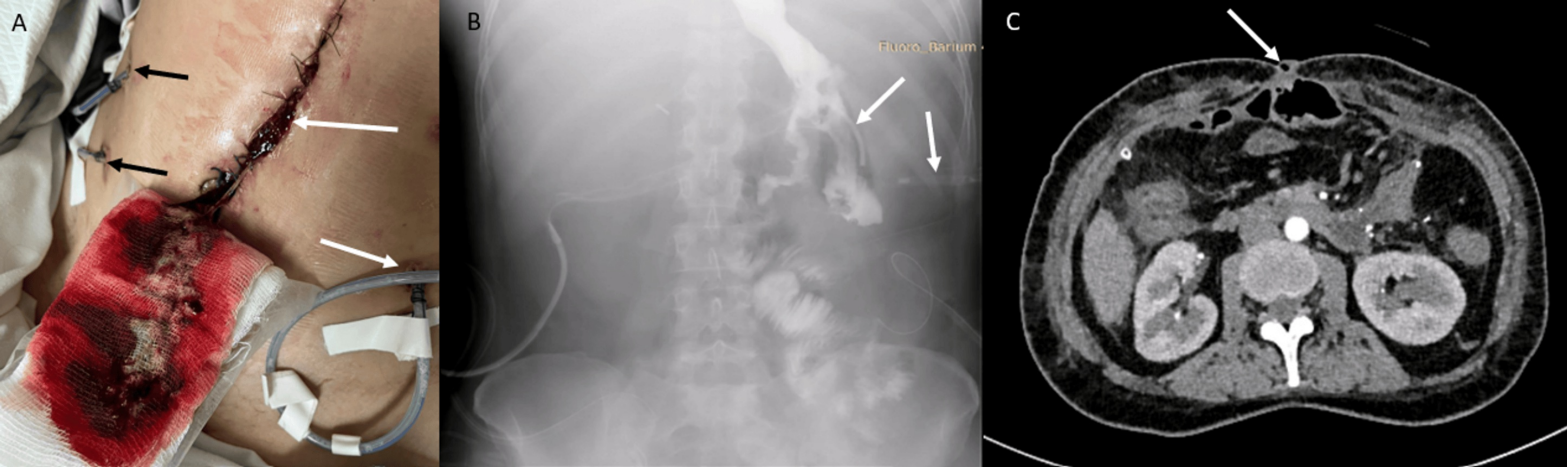
Multimodal evaluation of a postoperative gastrocutaneous fistula. (A) External abdominal view demonstrating surgical wound dehiscence with active drainage through cutaneous fistulous tracts (white arrows) and surgical drains in situ (black arrows). (B) Upper gastrointestinal contrast study showing extraluminal leakage of contrast from the gastric pouch, consistent with a gastrocutaneous fistula (white arrows), with two drains visible at the time of imaging due to interval removal of one of the previously placed drains. (C) Contrast-enhanced abdominal computed tomography confirming a transmural defect of the anterior gastric wall with communication toward the abdominal wall (white arrow), compatible with enterocutaneous fistula formation, corresponding to the later stage of clinical management.

After stabilization in the intensive care unit, the patient developed three fistulous tracts: two guided by surgical drains and one with spontaneous drainage of intestinal content through the surgical wound (Figure 1A), corresponding to the initial clinical manifestation of the gastrocutaneous fistula. During the patient’s clinical evolution, adjustments in the number and positioning of abdominal drains were performed as part of ongoing management. These findings were subsequently confirmed by an esophagogastrointestinal transit study, which demonstrated contrast leakage compatible with a gastrocutaneous fistula (Figure 1B), while a contrast-enhanced tomography revealed the presence of an enterocutaneous fistula (Figure 1C), representing the diagnostic stage of the transmural defects.

In this context of persistent sepsis, nutritional deterioration, and the presence of multiple complex fistulous tracts, the patient was referred to a third-level hospital for advanced endoscopic management.

**Figure 2:**
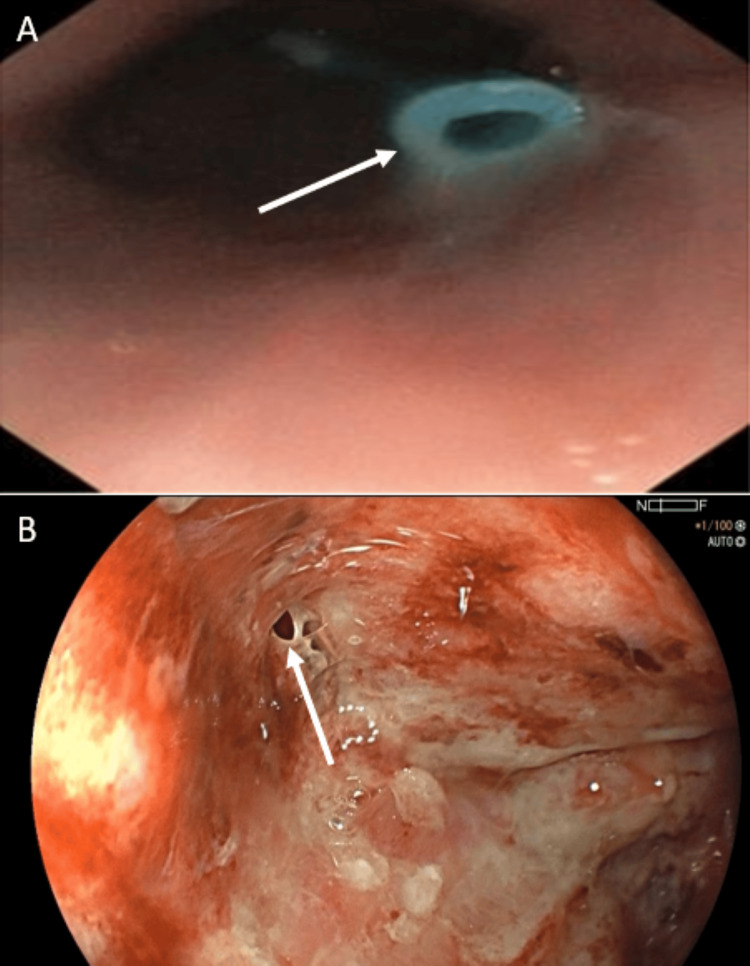
Endoscopic visualization of fistulous defects prior to modified endoscopic vacuum therapy. (A) Upper gastrointestinal endoscopic view demonstrating an internal fistulous orifice within the gastric pouch (white arrow). (B) Endoscopic image showing transmural defect with communication to an extraluminal cavity (white arrow), consistent with complex post-bariatric gastrocutaneous fistula. Surrounding mucosal inflammation and granulation tissue are evident.

Upon admission to the gastroenterology service, physical examination revealed abdominal tenderness without signs of peritoneal irritation. The patient exhibited signs of malnutrition and persistent abdominal drainage. Laboratory evaluation demonstrated leukocytosis with marked neutrophilia, thrombocytosis, elevated inflammatory markers, mild normocytic anemia, and hypokalemia, consistent with an active inflammatory and septic process in the setting of postoperative complications (Table 1). First diagnostic endoscopy was performed, confirming the presence of three transmural defects corresponding to a gastrocutaneous fistula, an enterocutaneous fistula, and a pancreatocutaneous fistula, which represent the endoscopic diagnostic stage of the disease prior to therapeutic intervention (Figure 2).

**Table 1:** Laboratory findings at admission. INR: international normalized ratio; aPTT: activated partial thromboplastin time

Parameter	Result	Reference Range
White blood cells (×10^3^/µL)	15.23	4.0-10.0
Neutrophils (%)	82.4	40-75
Hemoglobin (g/dL)	11.1	12-16
Hematocrit (%)	35.0	36-46
Platelets (×10^3^/µL)	581	150-400
Prothrombin time (s)	10.1	9-13
INR	1.07	0.8-1.2
aPTT (s)	29.1	25-35
Urea (mg/dL)	18.1	15-40
Creatinine (mg/dL)	0.33	0.6-1.2
C-reactive protein (mg/L)	35.7	<5
Sodium (mEq/L)	138.5	135-145
Potassium (mEq/L)	2.99	3.5-5.0

Given the complexity of the clinical scenario, the implementation of modified EVT was decided. For this purpose, an external modified EVT system was constructed using conventional hospital supplies (Appendices A, B), including a nasogastric tube, gauze, and antimicrobial dressing. Different system configurations were used, adapted to the anatomical characteristics of the fistulous tracts (Appendix C).

**Figure 3:**
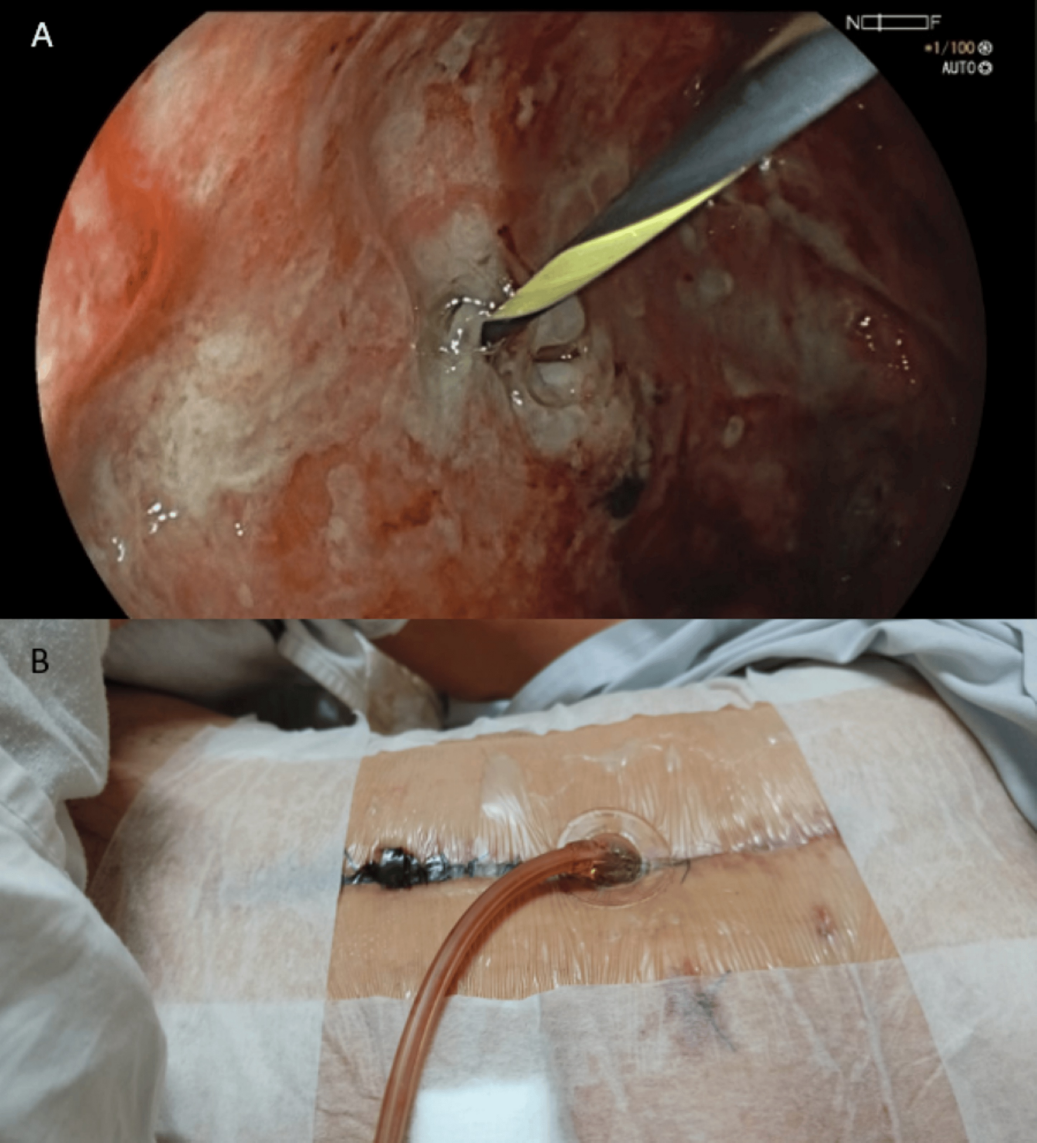
Endoscopic cannulation and external placement of a modified endoscopic vacuum therapy system. (A) Upper gastrointestinal endoscopic view demonstrating identification and cannulation of the internal fistulous orifice using a guidewire to facilitate placement of the modified endoscopic vacuum therapy device. (B) External abdominal view showing the modified endoscopic vacuum therapy system exiting through the cutaneous fistulous tract and secured at the skin level, allowing continuous negative-pressure drainage.

During the first endoscopic procedure, the gastrocutaneous fistula was identified and treated, and placement of the external modified EVT system was performed by passing a hydrophilic guide from the external fistulous orifice toward the residual gastric cavity (Figure 3A), representing the initial therapeutic stage of EVT. During this same procedure, distal jejunal stenosis was observed, which prevented passage of the conventional endoscope; therefore, a second intervention using an ultrathin endoscope was scheduled. Simultaneously, external modified EVT systems were placed in the pancreatocutaneous fistula, and a wall vacuum-assisted closure (VAC) system was placed in the enterocutaneous fistula (Figure 3B).

**Figure 4:**
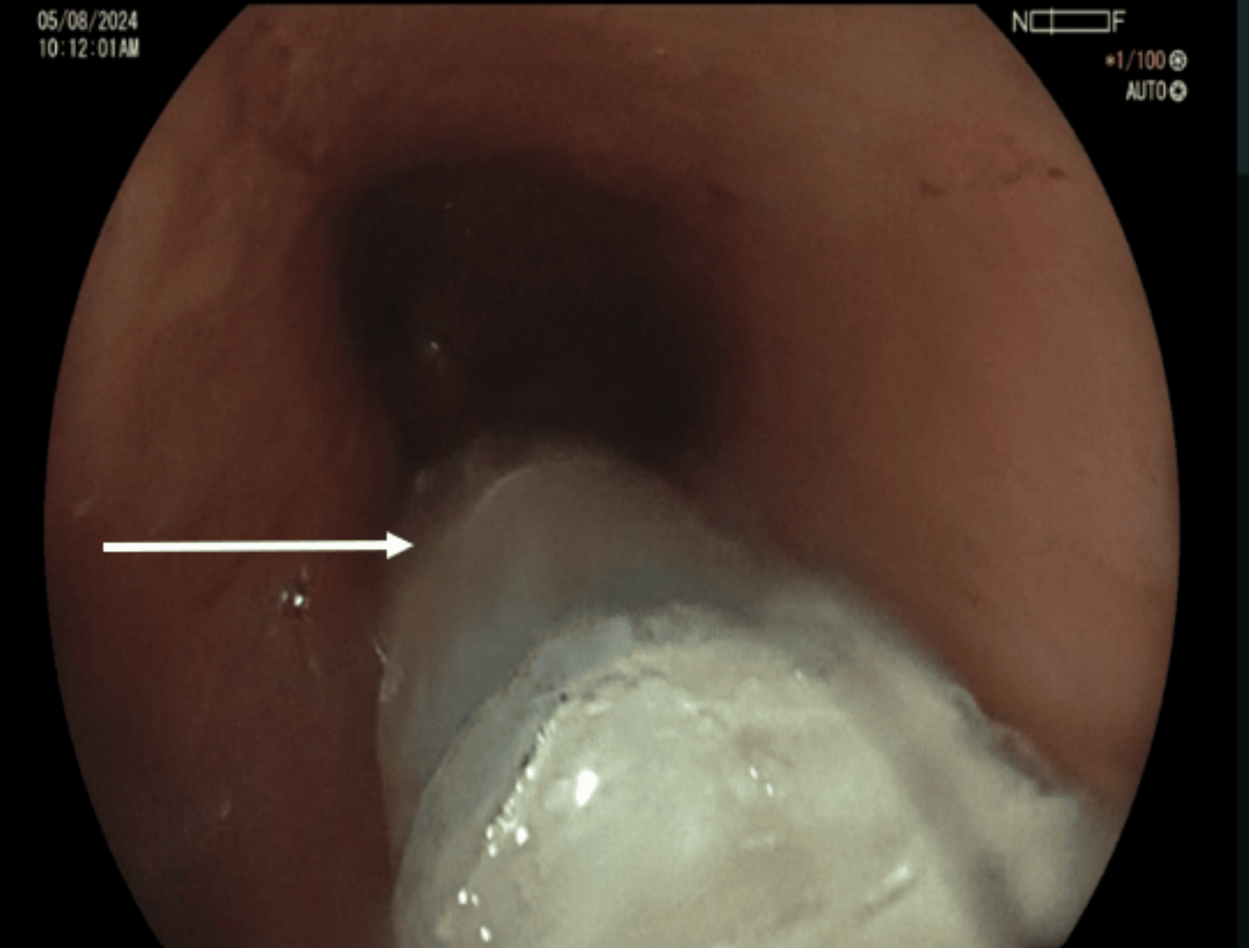
Intraluminal positioning of a modified EVT system. Upper gastrointestinal endoscopic image showing intraluminal placement of the modified EVT device within the gastric pouch (white arrow). EVT: endoscopic vacuum therapy

During the second upper digestive endoscopy performed with an ultrathin endoscope, it was possible to pass through the stenotic area and visualize the jejunum, allowing placement of an internal modified EVT system at the gastrocutaneous fistula site, representing the second therapeutic stage of EVT after endoscopic access through the jejunal stenosis (Figure 4).

The patient remained connected to the external modified EVT system for approximately one month and to the internal system for one week, both under continuous suction at 125 mmHg. During treatment, periodic clinical and endoscopic reassessment was performed to evaluate tissue granulation and verify the appropriate positioning of the vacuum system. Device exchanges were carried out according to clinical evolution and endoscopic findings, approximately once per week, until progressive collapse of the fistulous tracts and mucosal healing were observed. Broad-spectrum antibiotic therapy was maintained according to microbiological results and clinical response.

**Figure 5:**
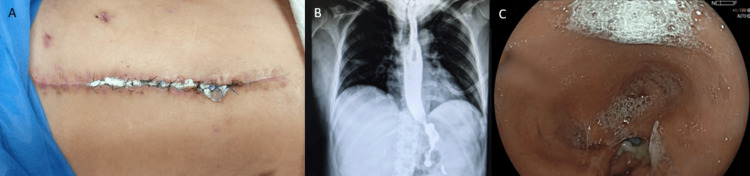
Clinical, radiologic, and endoscopic follow-up after modified endoscopic vacuum therapy. (A) External abdominal view demonstrating closure of the previous fistulous tract with appropriate healing of the surgical incision and absence of active cutaneous drainage. (B) Follow-up upper gastrointestinal contrast study showing normal passage of contrast through the esophagus and gastric pouch without evidence of extraluminal leakage. (C) Endoscopic view of the gastric pouch demonstrating closure of the previous fistulous orifice with mucosal healing at the prior defect site.

Progressive clinical improvement was observed during therapy, with gradual reduction of external drainage and resolution of the septic condition as the EVT system promoted progressive closure of the fistulous tracts. After 29 days of EVT therapy, progressive closure of the gastrocutaneous, enterocutaneous, and pancreatocutaneous fistulous tracts was achieved. Closure was confirmed clinically after device removal (Figure 5A), radiologically by control gastrointestinal transit study (Figure 5B), and endoscopically with absence of fistulous communication (Figure 5C).

During hospitalization, progressive clinical and laboratory improvement was observed. Follow-up laboratory tests showed normalization of leukocyte count and neutrophil percentage, improvement in hemoglobin and hematocrit levels, and correction of electrolyte imbalance. Inflammatory markers demonstrated a decreasing trend, and procalcitonin levels were within the normal range (Table 2). This clinical improvement paralleled the progressive closure of the gastrocutaneous, enterocutaneous, and pancreatocutaneous fistulous tracts achieved with EVT therapy. After one month of hospitalization, the patient was discharged in good clinical condition with adequate tolerance to oral intake.

**Table 2:** Laboratory findings before discharge.

Parameter	Result	Reference Range
White blood cells (×10^3^/µL)	8.30	4.0-10.0
Neutrophils (%)	57.3	40-75
Lymphocytes (%)	34.5	20-45
Hemoglobin (g/dL)	12.9	12-16
Hematocrit (%)	40.1	36-46
Platelets (×10^3^/µL)	438	150-400
Glucose (mg/dL)	102	70-110
Urea (mg/dL)	30.3	15-40
Creatinine (mg/dL)	0.45	0.6-1.2
C-reactive protein (mg/L)	27.1	<5
Procalcitonin (ng/mL)	0.04	<0.05

At the four-week outpatient follow-up, no clinical recurrence or signs of new leakage were observed, and the patient maintained adequate tolerance to oral intake. She reported progressive improvement in abdominal pain and overall functional recovery following removal of the vacuum therapy systems. The patient expressed satisfaction with the minimally invasive approach and with avoiding additional surgical interventions.

## Discussion

Leaks, fistulas, and other transmural defects that occur after bariatric surgeries are complications associated with sepsis, malnutrition, increased hospital stay, and an increased need for reinterventions, as was presented in the described case. In this context, endoscopic therapies have become the most widely used techniques with the best results by allowing control of the infectious focus, effective drainage, promoting granulation tissue formation, and closure of tissue defects, reserving surgical interventions in case of therapeutic failure of EVT. Currently, it is indicated that the endoscopic technique should be individualized according to the type of defect (leak vs. fistula), the time of evolution (acute vs. chronic), the presence of cavities or collections, the caliber of the fistulous tract, tissue perfusion, and the clinical/nutritional status of the patient [17,18].

In the present case, the patient had a complex clinical course, including multiple previous bariatric procedures, revision surgeries, sepsis, and the development of multiple fistulous tracts (gastrocutaneous and enterocutaneous), which were confirmed through various diagnostic modalities, including gastrointestinal transit studies, computed tomography, and endoscopy. These characteristics that occurred during the patient’s evolution and clinical picture are associated with a worse prognosis and a higher failure rate of traditional surgical strategies, particularly when there is extensive inflammation, contaminated cavities, and fistulous tracts. Therefore, an approach that combines continuous drainage of debris, local infection control, and that in turn promotes progressive tissue approximation would be considered the most effective strategy given the clinical scenario [17,18].

EVT is currently considered one of the most effective strategies for the management of upper gastrointestinal transmural defects, including those generated postoperatively, through multiple mechanisms of action that include drainage of exudate, reduction of bacterial load, reduction of local edema, improvement of tissue perfusion, promotion of granulation tissue formation, and stimulation of progressive contraction of the wall defect. A meta-analysis published in 2022 that sought to determine the efficacy of EVT for the treatment of post-bariatric surgery leaks and fistulas demonstrated a success rate for closure of these defects of 87.2%, with a low rate of moderate adverse events, with a 6% incidence [19].

Although EVT has demonstrated its clear superiority over invasive surgical techniques, its widespread application in healthcare systems is strongly linked to the economic aspect due to the availability of the devices required for this type of therapy. Faced with this difficulty that would considerably increase public health costs, proposing accessible modifications to the original system with a similar level of efficacy becomes a promising alternative that can be reproducible in multiple scenarios. The system used in the patient with the use of nasogastric tubes, gauze, and an adhesive/antimicrobial dressing demonstrated technical feasibility and may constitute a solid basis to adapt EVT in centers where access to commercial kits is limited [12,15].

In the case of the patient, a combined strategy of modified external EVT for control of the fistulous tract and continuous drainage was applied, and modified internal EVT when distal endoscopic access was achieved with an ultrathin endoscope after identifying jejunal stenosis. This strategy is consistent with current recommendations on EVT that propose choosing between intraluminal or intracavitary positioning (or combinations of both), according to the anatomy, the fistulous tract, and the presence or absence of a collection [17,18].

The favorable evolution of the patient, associated with clinical and imaging closure of the fistulous tracts after 29 days of treatment, is consistent with the timelines described in EVT literature, where the total duration of treatment varies according to the size of the defect, infection control, the need for system exchanges, and the recovery of the ability to ingest food. It is important to note that the initiation of tolerance to enteral diet is fundamental to determine favorable clinical evolution after EVT therapy, considered a major determinant of healing and associated with a reduction in the risk of complications [18,20].

When comparing EVT with other endoscopic techniques for the closure of wall defects including SEMS, internal drainage with double pigtail, septotomy, and endoscopic sutures, it suggests that EVT is one of the therapeutic options with the best results, comparable to other internal drainage strategies; however, studies comparing these types of interventions are quite heterogeneous among themselves; furthermore, randomized clinical trials are not available to establish clear algorithms for the management of these defects; therefore, the choice of technique depends on the type of defect and the experience of the healthcare unit in each technique [17,21].

Although EVT is a therapy that has demonstrated clear efficacy, it is not exempt from adverse events, including pain due to the presence of the tubes, bleeding, system migration, stenosis, and, much less frequently, perforation or worsening of the defect in cases where the presence of necrosis or ischemia has not been adequately identified. With the aim of avoiding and reducing the rate of complications, scheduled serial exchanges and evaluation with contrast imaging studies prior to patient discharge are considered important recommendations when using this technique [13,20].

This clinical case also stands out by demonstrating the feasibility of an internal and external modified EVT system assembled with supplies of habitual use in hospital units, which allowed successful closure of multiple fistulas after several bariatric surgeries. The main value of this lies in demonstrating reproducibility with accessible materials, showing its usefulness in a complex clinical scenario, and highlighting the need for a stepwise approach: initial diagnostic endoscopy, adaptation of an ultrathin scope due to stenosis, and transition to an internal system.

The main limitation inherent to this case report is its methodological nature, as it does not allow demonstration of causality nor comparison with other therapeutic modalities. Quantitative parameters such as serial measurements of fistula tract diameter or precise drainage volume were not systematically documented during routine clinical management, which represents a limitation of this report. Future studies incorporating standardized quantitative monitoring may provide a more objective assessment of treatment response. Additionally, although short-term clinical, radiological, and endoscopic resolution of the fistulous tracts was confirmed, longer follow-up would be necessary to evaluate potential late complications such as recurrence of the fistulas or luminal stenosis.

This case highlights that modified EVT may be a feasible and effective option for the management of complex post-bariatric fistulas in resource-limited settings when individualized according to anatomical and clinical characteristics.

## Conclusions

In conclusion, modified EVT using low-cost materials demonstrated a feasible and promising therapeutic option for the management of transmural defects following bariatric surgery. In this case, the technique allowed successful closure of multiple fistulous tracts in a clinically complex scenario, with confirmation of healing through clinical, radiological, and endoscopic evaluation. These findings suggest that adapted EVT systems constructed from commonly available materials may represent a practical alternative in selected patients, particularly in resource-limited settings. However, further studies and longer follow-up are required to better evaluate reproducibility, long-term outcomes, and the optimal role of this technique in the management of post-bariatric fistulas.

## Figures and Tables

**Figure 6:**
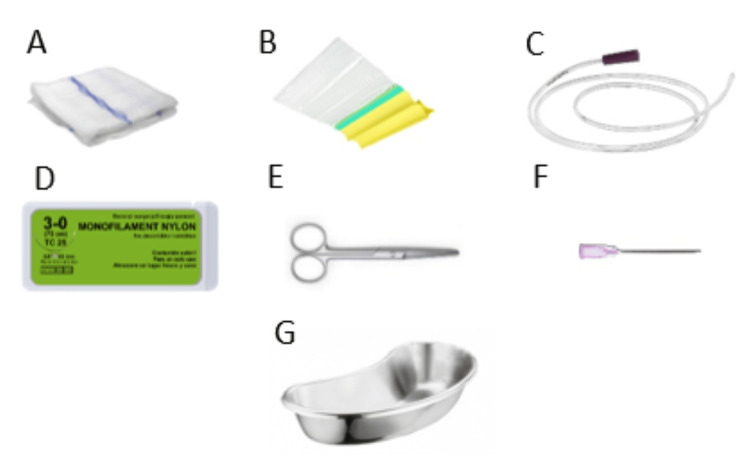
Materials required for the construction of a modified endoscopic vacuum therapy system. (A) Sterile gauze pad; (B) Antimicrobial incise drape; (C) Nasogastric tube (14-20 Fr); (D) Monofilament suture material; (E) Surgical scissors; (F) 18-gauge needle; (G) Stainless steel bowl for system integrity testing.

**Figure 7:**
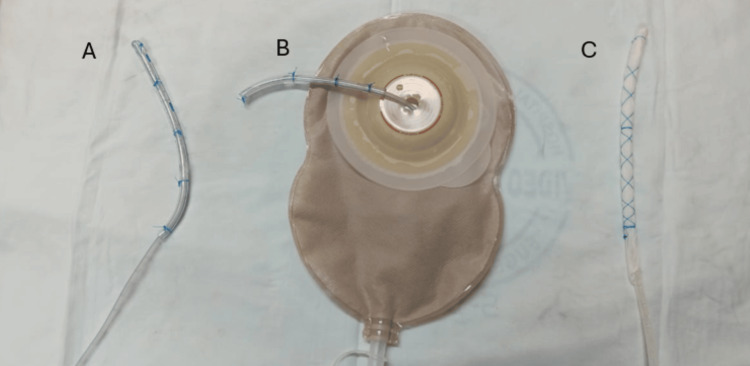
Variations of modified EVT systems. (A) Simple modified EVT system assembled with a fenestrated nasogastric tube and a gauze-drape covering. (B) Modified EVT system adapted to a cystostomy bag for controlled external drainage. (C) Tube-on-tube modified EVT configuration designed to enhance structural stability and suction distribution. EVT: endoscopic vacuum therapy

## Appendices

Appendix A

Appendix B

**Table 3:** Step-by-step assembly and placement of a modified endoscopic vacuum therapy system.

Step	Description
1	Create multiple fenestrations in the distal third of the nasogastric tube.
2	Wrap a sterile gauze pad around the fenestrated segment of the tube.
3	Cut the antimicrobial surgical drape to match the size of the fenestrated segment, covering the gauze completely.
4	Secure the assembly with monofilament sutures.
5	Perform multiple punctures through the drape using an 18-gauge needle to allow negative-pressure transmission.
6	Test the device in a bowl filled with water to verify system integrity and absence of leaks.
7	Place the device endoscopically (internal or external positioning according to defect anatomy).
8	Secure the internal device at the nasal level using adhesive fixation.
9	Secure the external device to the skin using sutures.
10	Connect the system to continuous suction at 125 mmHg and verify proper negative-pressure function.

Appendix C
